# Development of the first axillary in vitro shoot multiplication protocol for coconut palms

**DOI:** 10.1038/s41598-021-97718-1

**Published:** 2021-09-15

**Authors:** Hannes Wilms, Dries De Bièvre, Kevin Longin, Rony Swennen, Juhee Rhee, Bart Panis

**Affiliations:** 1 Laboratory of Tropical Crop Improvement, Biosystems, KU Leuven, Leuven, Belgium; 2 International Institute of Tropical Agriculture, Plot 15B Naguru East Road, Upper Naguru, Box 7878, Kampala, Uganda; 3 National Agrobiodiversity Center, RDA, Jeonju, Korea; 4 Bioversity International, Belgian Office at KU Leuven, Leuven, Belgium

**Keywords:** Plant biotechnology, Plant cell biology, Non-model organisms, Cytokinin

## Abstract

The coconut palm or “tree of life” is one of nature’s most useful plants and the demand for its fruit is increasing. However, coconut production is threatened by ageing plantations, pests and diseases. Currently, the palm is exclusively propagated via seeds, limiting the amount of planting material. A novel micropropagation method is presented, based on axillary shoot formation. Apical meristems of in vitro coconut seedlings are cultured onto Y3 medium containing 1 µM TDZ. This induces the apical meristem to proliferate through axillary shoots in ~ 27% of the initiated explants. These axillary shoots are seen as white clumps of proliferating tissue and can be multiplied at a large scale or regenerated into rooted in vitro plantlets. This innovative micropropagation method will enable the production of disease-free, high quality in vitro plantlets, which will solve the worldwide scarcity of coconut planting material.

## Introduction

Among the estimated 2600 living palm species^1^, the Coconut palm (*Cocos nucifera* L.) ranks as one of the most important ones. Since the palm’s domestication in the Pacific and Indian ocean^2^, it provided many Southeast Asian communities numerous commodities, such as food, shelter and drinks^3^. Because of its importance in the region, the palm gained a mythological status in Polynesian folklore^4^ and earned itself the nickname “the tree of life”. While the palm is used for many purposes nowadays, the global market is mainly focused on its fruits, as they are a source of oil, coconut water, coconut milk and fiber^3^. Since 1961, the production of coconuts has been steadily increasing, with plantations being set up all over the tropics, reaching 62.5 M tons in 2019^5^, and this trend is expected to continue.

However, coconut cultivation faces multiple challenges worldwide. Firstly, many plantations are over their economic lifespan^6^; indeed, when the coconut palm reaches the age of 40–70 years, depending on the cultivar, their production drops significantly^7^. Secondly, partially due to globalization, deadly diseases such as Lethal yellowing or Cadang-Cadang^8,9^ and pests such as the red palm weevil (*Rhynchophorus ferrugineus*) and rhinoceros beetle (*Oryctes rhinoceros* L.)^10^, have spread over multiple continents. Both challenges combined with a global increased consumption of coconut products cause a greater demand of high quality disease-free and -resistant plant material.

The supply of quality plant material is currently limited by the number of coconuts that one palm can produce, which ranges between 40 and 100 fruits a year^11^. Moreover, new plantings from seeds are always genetically diverse due to cross pollination, especially in the case of the tall-type coconuts. Such variation might cause the loss of desired characteristics, such as disease resistance or drought tolerance^12^. Seed propagation is the only method of reproduction since the palm’s domestication as this palm does not produce suckers or branches^13^. Forty years after the first attempt to micropropagate coconut tissue by Cutter and Wilson^14^, Verdeil and coworkers reported the first in vitro multiplication success via somatic embryogenesis (SE) in 1994^15^.

Following this report, researchers further developed SE in coconuts and applied it to a wide array of explants, such as immature inflorescences, plumules and zygotic embryos^16^. SE derived plantlets in general are, however, often prone to somaclonal variation^17^, causing unwanted off type clones. These effects are observed in other palm species^17,18^ such as oil palm; many SE derived trees suffer from “the mantled phenotype” reducing its fruit’s oil content^19^. Alternative propagation methods with lower risk of somaclonal variation are therefore required. Such alternatives fall into two main groups: multiplication by inducing adventitious meristems or by inducing axillary meristems^20^. Both methods, which are highly successful in many plant species, have not proven to be successful yet in the coconut palm.

Adventitious meristems originate from non-meristematic cells and are therefore created de-novo. These meristems can be induced from a wide variety of plant tissues, such as leaves, hypocotyls and roots of dicotyledonous plants (e.g., Brassica spp.; Malus domesticus; Helianthus annuus)^21–23^. In monocotyledonous plants this method proved to be more difficult^24,25^. It must be noted, however, that some ambiguity does exist in literature with respect to the use of term adventitious meristems, as some researchers also include the formation of meristems from pre-existing meristematic zones (in actual fact not de-novo) under this category. This is why such reports need to be carefully studied for their explant sources and definitions.

Axillary meristems are always induced from pre-existing meristematic cells and lead in vivo to the formation of branches or flowers. These meristems or meristematic zones often remain dormant under the influence of the apical meristem^26^, which limits the formation of multiple branches, or in the case of coconut, completely arrests the formation of new branches^13^. Such dormancy is controlled by different plant hormones families: cytokines, auxins, strigolactones and carotenoid-like plant hormones^26,27^. While all these hormones influence the outgrowth of axillary meristems, the main mechanism in determining branching behavior is the cytokinin—auxin balance. Auxins are produced in the shoot apical meristem and repress the underlying axillary nodes, while its antagonist, cytokinins, which are formed in the root tips, promote axillary shoots development. Changing the balance of these Plant Growth Regulators (PGR) near the axillary meristematic tissue can thus promote or repress the formation of new shoots^28^. One way to influence this balance is by removing the auxin source, often by removing, splicing or damaging the apical meristem. Alternatively cytokinins such as 6-Benzylaminopurine (BA), zeatine, kinetin or cytokinin-like PGR such as Thidiazuron (TDZ) and Forchlorfenuron (CPPU) can be added to the tissue culture medium^29^.

While the coconut palm is not producing side shoots or branches in vivo, the ability to produce axillary meristems is not completely lost since their presence is a prerequisite for flowering. Such “floral” axillary meristems could be sources of vegetative meristems, as, in other plants, it has already been shown that immature male flowers are able to revert from their regenerative state to a vegetative state^30^. Secondly, there have been sporadic observations in nature of branching coconut palms^13,31^. Both observations therefore open the possibility of clonal propagation via axillary shoot formation, a hypothesis that is tested in this study by manipulating the endogenous PGR balance.

## Results

### The effect of cutting in the presence or absence of TDZ on inducing proliferating meristems

**Figure 1:**
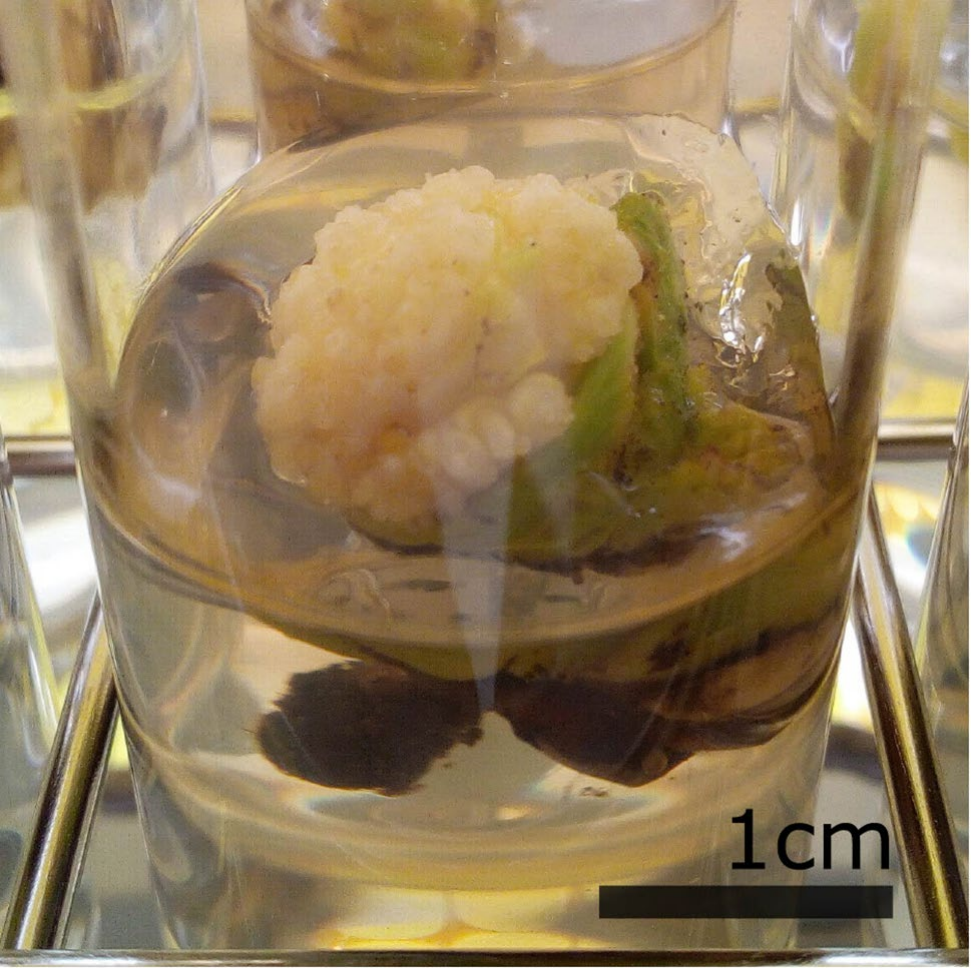
Proliferating meristems clumps of the MYD cultivar emerging from the centre of the coconut plantlets, 45 days after initiation.

**Figure 2:**
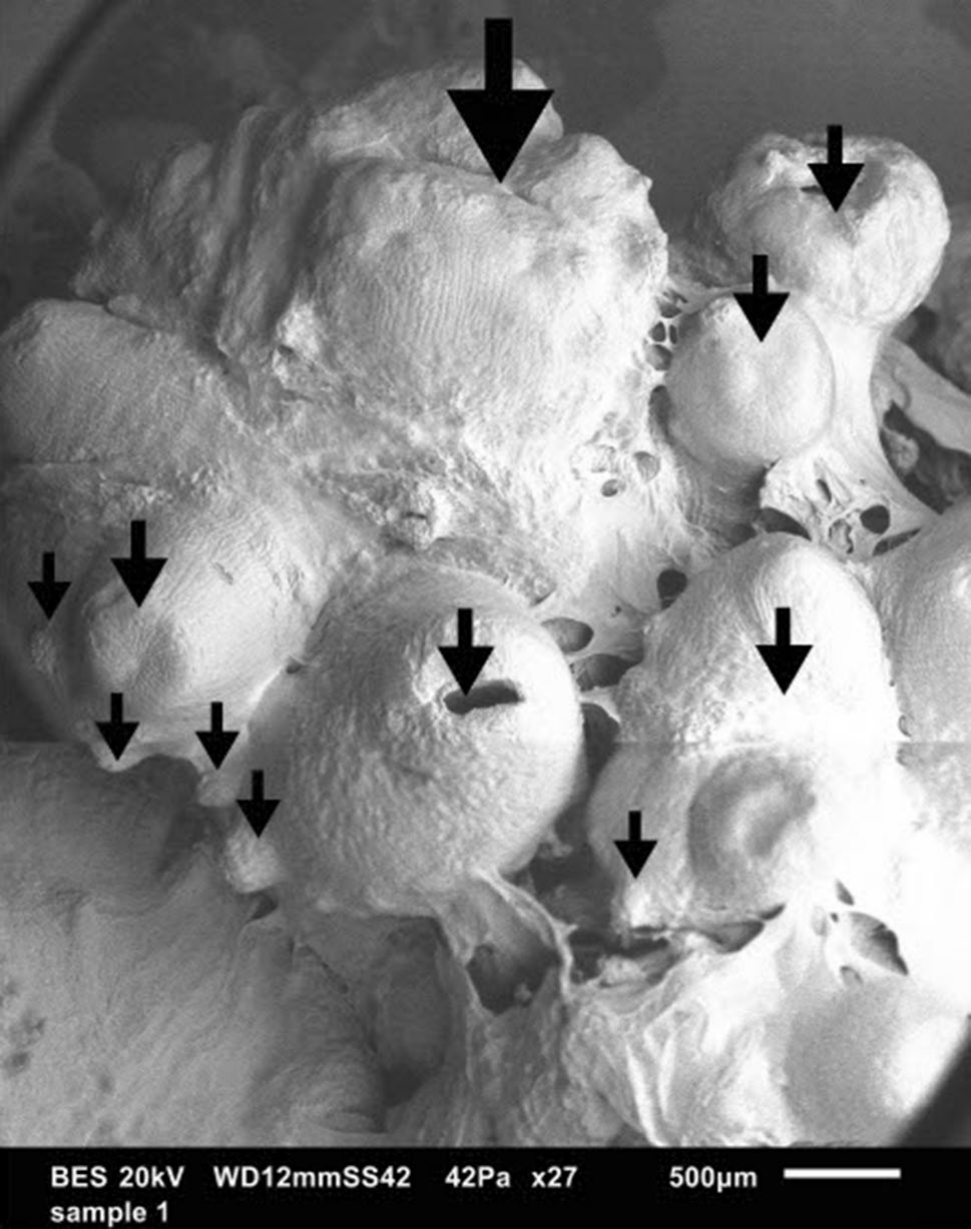
Scanning electron microscope view of the surface of a proliferating meristem clump. The central meristem can be seen in the middle and is, at the basis, surrounded by new (axillary) meristems The same axillary meristems are on their turn the basis, or new central meristem, for new axillary meristems. Meristems are annotated with an arrow. This figure was made by combining two separate scans of the same object (with permission of Bart Wilms).

Forty-five days after initiation, 36 of the 141 (25.5%) explants that were subjected to the “cut protocol” combined with culture on 1TDZ, proliferated by exhibiting a white enlarged meristematic zone with many multiplying meristems arising from the former center of the plantlet (Fig. 1). These meristematic zones always originated from only one of the two halves that were initiated; the other part blackened and died, irrespective of the culture medium. The plantlets of the two other treatments (“control treatment” + 1TDZ and “cut protocol” + Y40) did not show any signs of proliferation. The Scanning Electron Microscope (SEM) picture shows the formation of new meristems at the base of older meristems in a structured (spiral-like) pattern (Fig. 2), pointing at the axillary origin of the side meristems.

### The effect of different cytokinins or cytokinin-like PGRs on inducing proliferating meristems

All 3 tested compounds were able to induce meristematic proliferation after 45 days (Table 1), however only 1 µM TDZ and 10 µM CPPU resulted in a proliferation rate significantly higher than treatments resulting in no proliferation. It was also observed that 8.3% of plantlets that were transferred to 100 µM BA produced proliferating meristems but also that 41.7% died, which is significantly higher than the mortality rate of the other treatments (6.3%).

**Table 1:** The reaction of MYD plantlets 45 days after initiation on different concentrations of PGR.

PGR	Concentration (µM)	Plantlets Initiated	Meristematic proliferation	Died after initiation
TDZ	0.1	24	0 (0%)a	2 (8.3%)a
TDZ	1	22	5 (22.7%)b	1 (4.5%)a
BA	1	24	0 (0%)a	0 (0%)a
BA	10	24	0 (0%)a	0 (0%)a
BA	100	24	2 (8.3%)a	10 (41.7%)b
CPPU	0.1	20	0 (0%)a	1 (5.0%)a
CPPU	1	21	1 (4.8%)a	4 (19.0%)a
CPPU	10	23	4 (17.4%)b	2 (8.7%)a

Different letters in a same column signify statistical differences (α = 0.05).

### The effect of the “meristem protocol” vs “cut protocol”

After 45 days, the meristem protocol induced proliferating meristems in 9 out of 48 (18.8% ) initiated explants, while the, less time consuming, cut method, resulted in 15 of 49 (30.6%) of the explants reacting. Both results, however, did not differ statistically significant.

### Proliferation of six cultivars

**Figure 3:**
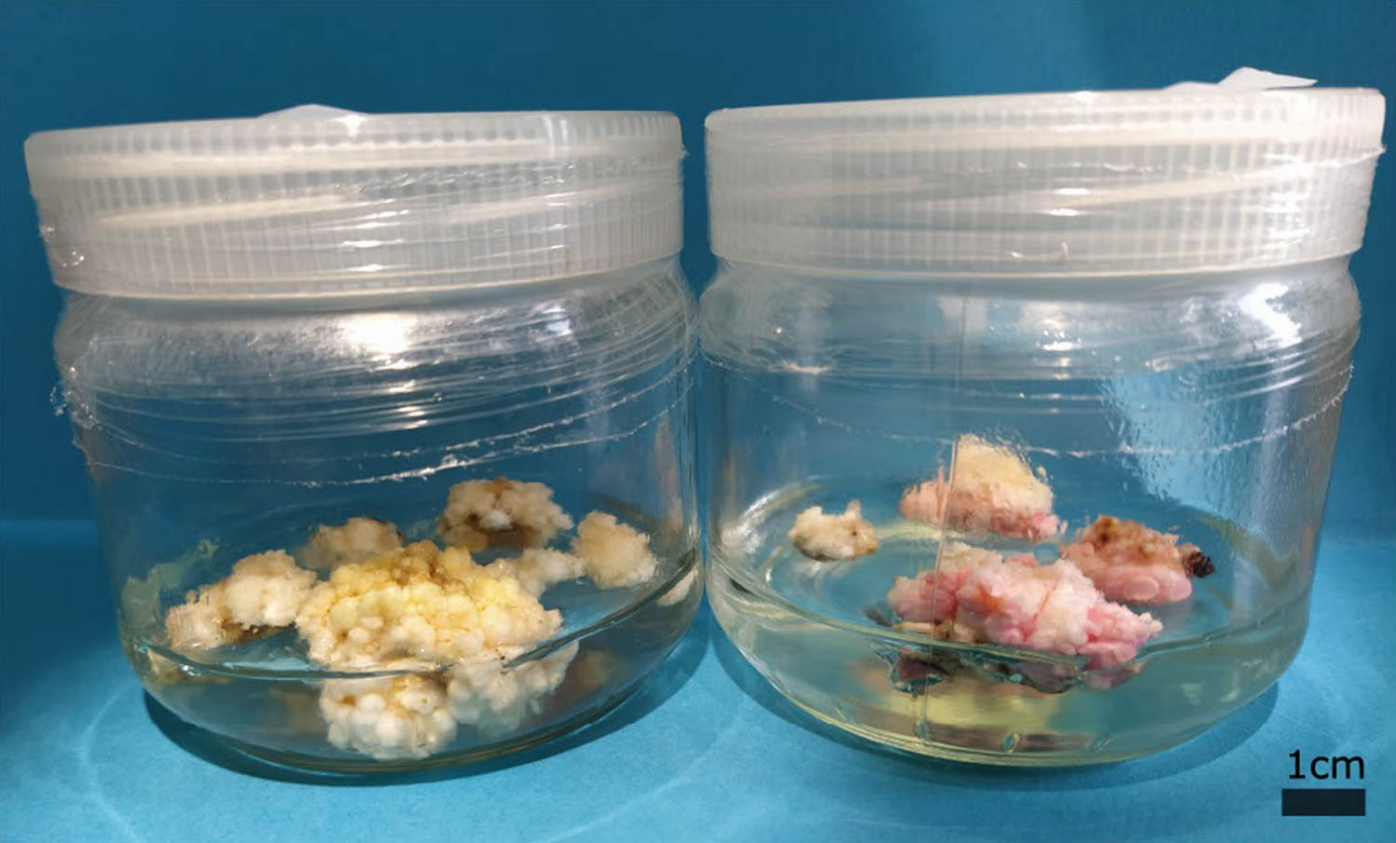
Proliferating meristem cultures originating from 2 different cultivars. Left MYD scalps, cultured on 1TDZ medium for 3 years, with the “normal” coloration, Right MVT scalps having a pink coloration.

No significant differences were observed between the responses of the six cultivars (Table 2). All showed comparable proliferation (average 27.1%) and mortality (average 13.5%) rates after 45 days. Also the type of the coconut variety (tall versus dwarf) did not influence their reaction. We observed that the proliferating material of one of the cultivars, MVT, showed a pink coloration (Fig. 3) but no other disfigurations or colorations were observed.

**Table 2:** The reaction (with standard deviation) of the six different cultivars on 1TDZ medium, 45 days after the cut treatment.

	Cultivar	Plantlets	Meristematic proliferation	Died after initiation	Meristematic proliferation
		Initiated	(Percentage ± StD)	(Percentage ± StD)	(Percentage ± StD)
*Dwarf*	CATD	72	22 (30.6 ± 14.6%)a	8 (11.1 ± 6.3%)a	58 (26.9 ± 12.0%)a
	EGD	72	21 (29.2 ± 14.4%)a	10 (13.8 ± 13.4%)a	
	MYD	72	15 (20.8 ± 8.3%)a	5 (6.9 ± 2.4%)a	
*Tall*	LAGT	72	14 (19.4 ± 2.4%)a	19 (26.4 ± 15.8%)a	58 (27.1 ± 12.0%)a
	MVT	72	24 (33.3 ± 7.2%)a	10 (13.8 ± 6.4%)a	
	WAT	70	20 (28.6 ± 19.1%)a	6 (8.6 ± 8.3%)a	
	Total	430	116 (27.1 ± 11.6%)	58 (13.5 ± 10.5%)	

As there were no statistical differences an extra row with the combined reaction rate was added.

Different letters in a same column signify statistical differences (α = 0.05).

### Subculture of proliferating material

The proliferating meristem clumps could be maintained, in their proliferating state, on the 1TDZ medium over the course of 3 years (Fig. 3) provided they were subcultured monthly, as the tissue tended to become brown if left on the same culture medium for longer periods. During their maintenance, we observed that the size of each meristem clump almost doubled every month.

### Regeneration

**Figure 4:**
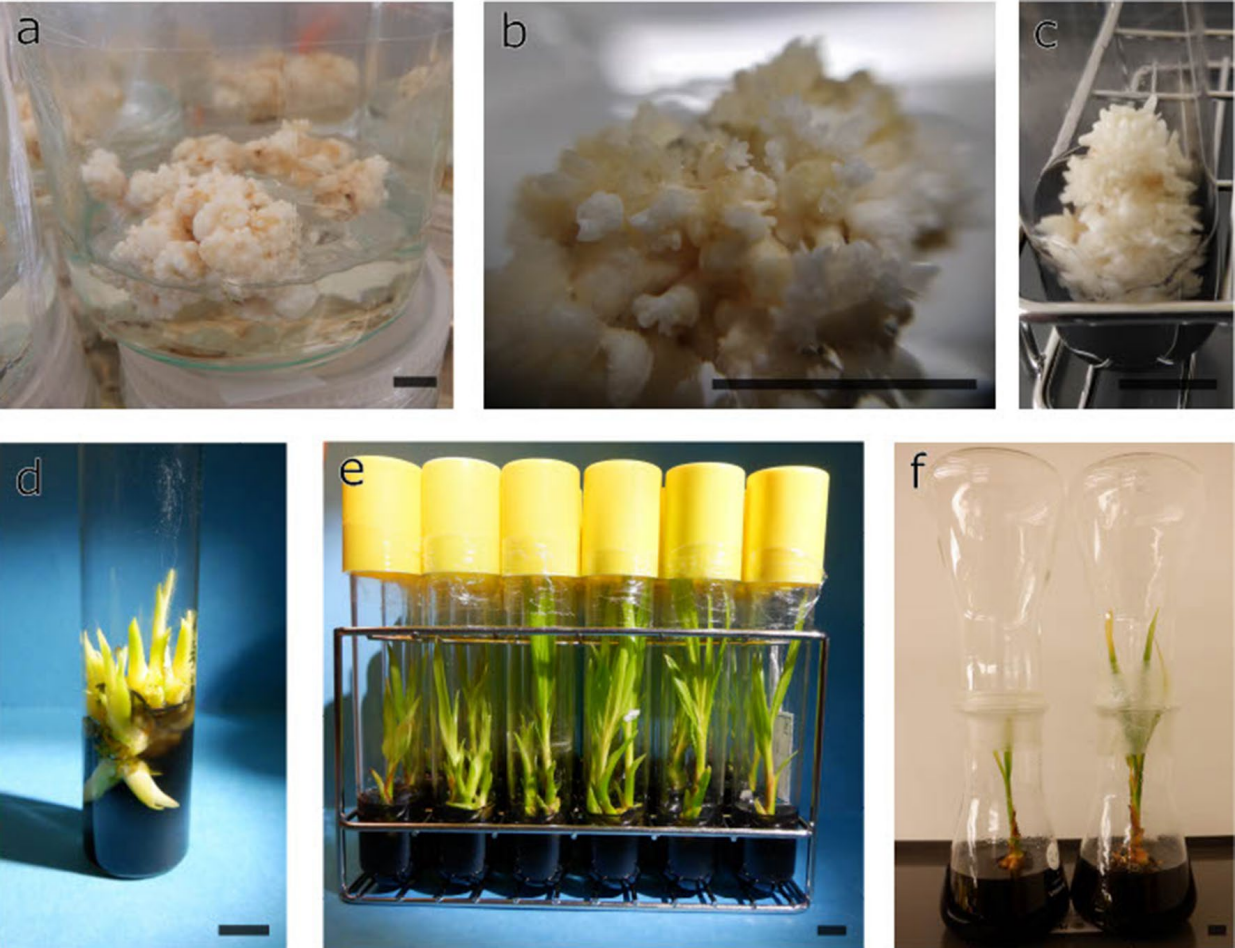
The regeneration process of proliferating coconut meristems over a 10 month period. (**a**) Proliferating meristems on 1TDZ medium. (**b**) Meristem clump after 1 month of culture on TDZ free medium. (**c**) Meristem clump after 2 months of culture on TDZ free medium. (**d**) 4 month old cluster of plantlets derived from meristem clumps. (**e**) 8 month old shoots derived from meristem clumps. (**f**) Rooted shoots, left plantlet derived from meristem clumps, right control, both ready for transfer to the greenhouse. The black bars each represent 1 cm.

**Figure 5:**
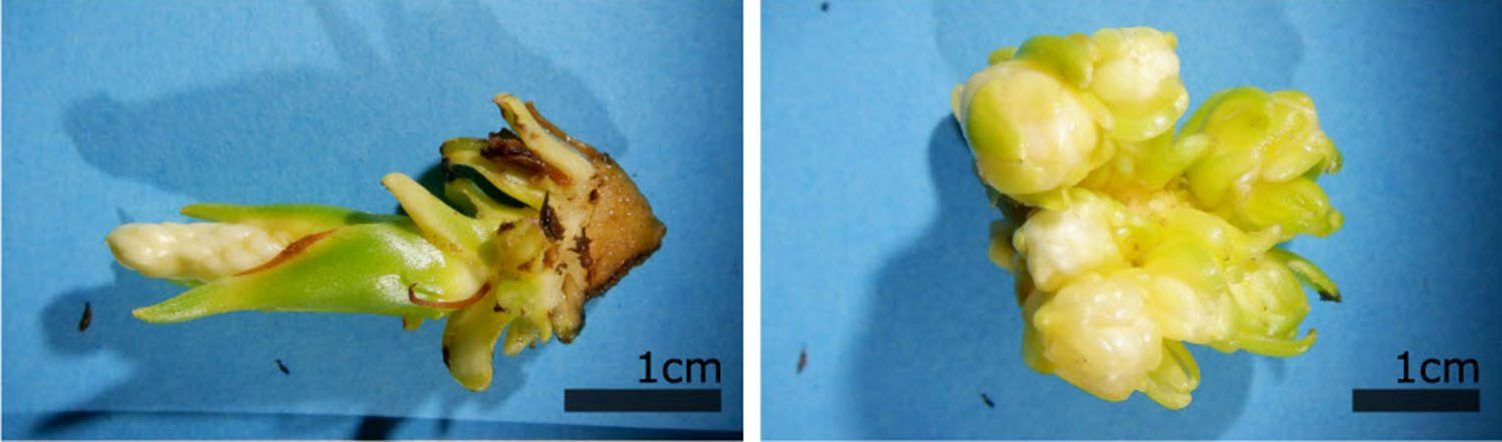
Two types of flowerlike structures observed during the regeneration process. Left, cone-like outgrowth resembling immature male spadix covered with scale-like leaves. Right, ball-like structures resembling immature female flower structures.

Over the course of 2 years, samples were regularly isolated from the continuous proliferating meristems for regeneration. Each regeneration attempt, resulted in rooted in vitro plantlets, regardless if the samples were taken directly after initiation or after two years of subculturing on the 1TDZ proliferation medium. Eight to 70% of the meristems present per ~ 0.8cm^2^, measured at the base, sized proliferating clumps, depending on the clone, regenerated into shoots that could be rooted (Fig. 4). As such, 5 to 18 cloned plantlets were produced on Y40 AC medium per clump after six to eight months (Fig. 4). The remaining 30 to 92% meristems per clump that did not develop into normal shoots showed flowerlike structures (Fig. 5) or became necrotic and died.

## Discussion

The development of a reliable and fast clonal multiplication technique for coconut palms is essential to meet the increasing industry demand for quality planting material. This would allow breeders and nurseries to bring large numbers of cloned plantlets onto the market, that are free of diseases and have moreover superior characteristics. In addition, this would also greatly help conservation efforts of coconut genetic resources through in vitro conservation and cryopreservation^32^. For more than 50 years, the search for an efficient clonal propagation method for coconut has been the subject of many studies. This has initially led to the development of various SE protocols^33^. However, somatic embryogenesis still bears major challenges that need to be resolved^17,19,34^ before it can be used in a commercial setting. This study thus focuses on the development of an alternative micropropagation method, that would mitigate some of the problems associated with SE such as somaclonal variation, low induction rates and cultivar dependence^19,32,35^.

In our first experiment, we tested the hypothesis that latent axillary meristems could develop into shoots provided the apical dominance of the apical shoot was eliminated. A first method consisted of cutting the explant, that contains one apical meristem, in two equal pieces. This cut would either damage the apical meristem irreversibly, provided it went exactly through the apical meristematic dome or remove the meristem from one of the two pieces. The second method involved the addition of a cytokinin-like PGR (TDZ) to the culture medium. In banana, another monocot species, TDZ already proved to be very powerful in reducing the apical dominance resulting in highly proliferating meristem clumps^36^. While these two procedures, combined or alone, have been successfully applied in many plant species, both monocotyledons and dicotyledons, e.g. banana^36^, ensete^37^ & pitaya^38^, these techniques proved to be ineffective in coconut. Sisunandar and coworkers developed an embryo incision method that when combined with the addition of cytokinins in the medium, resulted in only two cloned plantlets per embryo^39^. Our results significantly differed from this report in two ways; firstly the plantlets that were cut in two, both in the presence and absence of TDZ, always resulted in the death of one part. Secondly, in 25% of the plantlets that were treated with the “cut + TDZ” treatment, a new structure appeared, i.e. a cluster of meristems, somewhat comparable to a cauliflower structure.

As this meristem cluster appears only in one of the two halved shoots, the apical meristem is likely the meristem cluster’s source. This statement is also supported by the location of the meristem cluster, which is in the former center of the explant (Fig. 1). In case the meristem cluster would have originated from a somatic cell (de novo organogenesis) or from a “sleeping” axillary meristem, in the absence of the apical dominance, both halves should have formed new meristems.

While these meristem clusters share some general visual similarities with the structures that have previously been generated through coconut SE, such as their white-opaque-yellow lobbed exterior^40,41^, there are key differences between the two tissues. The meristem clusters have a more uniform white color and are not as transparent. Also they are structurally more orderly arranged (Fig. 2), compared to the somatic embryo clusters. This difference can be explained by their different origins; somatic embryos do appear more randomly, while the organized pattern observed in the meristem cluster is caused by the formation of new axillary meristems at the basis of the older apical meristems. Such structures are also reported in two other monocotyledonous species; date palm bud cultures obtained by Sidkey^42^ and banana shoot clusters by Strosse and coworkers^36^.

We observed that 100 µM BA was also able to induce the same structures as with much lower concentrations of TDZ. This observation strengthens the hypothesis that TDZ, along with its direct cytokinin-like activity, acts upon multiple pathways in the plant^29^ as well as confirming earlier reports claiming that TDZ has a stronger cytokinin-like activity compared to adenine based cytokinins such as BA, kinetin and zeatin^36,43^. Moreover, the high BA concentrations needed to induce meristem clump formation in coconut proved to be more toxic compared to TDZ. We also observed (results not shown) that prolonged culture of the meristem clumps on 100 µM BA leads to severe tissue necrosis in line with other reports^36,44^.

The cytokinin like activity of TDZ^45^ is explained through the direct interaction of TDZ with cytokinin receptors^46^ and the inhibition of endogenous cytokinin degradation^47^. CPPU, another phenylurea derivative, which was found to be a more potent inhibitor of cytokinin degradation compared to TDZ in maize^47^, resulted in our study in a lower amount of meristem clumps being formed, when using similar concentrations. This suggests that a direct interaction might be TDZ’s main course of action in coconut, as otherwise the explants treated with CPPU would result in a similar or even higher proliferation rate. However, earlier comparisons between CPPU and TDZ show that their relative potency might be plant and tissue dependent^48,49^.

Since no significant difference in frequency of axillary shoot formation was observed between the “meristem” and “cut” method, shoot proliferation originates from the intact apical meristem. The facilitated uptake or transport of PGRs to the apical meristem are thus the main factors in successfully inducing proliferating meristems. The full sized in vitro plants, containing many “buffering” tissue layers, were not successfully induced, which was probably due these layers diluting or restricting the PGR flow to the meristem.

The meristematic induction rate of the six different cultivars under investigation, did not significantly differ and was on average 27%. As these cultivars represent the genetically distinct tall and dwarf types, it is expected that the protocol is applicable to more if not most coconut palms. It would therefore also be worthwhile to investigate if this method would be applicable other members of the palm family (*Arecaceae*) as in vitro shoot multiplication protocols are also needed in date and oil palm industries.

This successful regeneration of proliferating meristems into rooted in vitro plantlets was obtained even after 2 years of subculture and multiplication. This proves that the morphology of the proliferating meristems is maintained for multiple years resulting in a virtually unlimited source of clonal plant material.

While each regenerated meristem clump produced 5 to 18 normal in vitro plantlets, after 7 to 8 months, on average 52% of the meristems grew into flowerlike structures. The latter resemble the coconut flower initiation stages described by Krisanapook and coworkers^50^ and are probably initiated by TDZ as this PGR is known to induce flowering in some plants^51^. While these structures can be regarded as undesirable, they could be of potential use for speed breeding practices. If for example pollen or ovaries could be produced from these immature flowers, breeding cycles could be shortened significantly. Currently it takes 10 years from seed to assessment^52^. Other researchers have already attempted to induce flowering in oil and date palm with varying results^53,54^. Masmoudi-Allouche and coworkers were able to induce in vitro date palms to develop ovaries, however, to date these findings in the palm family are not yet applied in speed breeding^55^.

While in this report in vitro multiplication was initiated from zygotic embryos, this technique has the potential to be applied on fully characterized mature palms. In other palm species it has been shown that apical shoots isolated from field grown palms (off-shoots) could be used^56^ as starting material for clonal propagation, and for bananas (monocot) it has been proven that even immature male inflorescence could be used^30^. The latter technique would have the advantage of being non-destructive. However, this still needs to be experimentally confirmed.

During the next few years, regenerants derived from our multiplication protocol will be verified under field conditions. It is known that clonal propagation of oil palm through somatic embryogenesis often results in a high frequency of the unwanted mantled phenotype^19^, only visible during fruiting. However, we believe that by using a multiplication protocol based on axillary shoot multiplication, the chance to obtain such off types is considerably reduced.

## Conclusions

The “cut” treatment, where the plantlets are vertically cut in two at the middle, on in vitro coconut plantlets combined with exposure to 1 µM TDZ in the culture medium, resulted in the meristematic proliferation of the apical meristem in up to 33.3% of the initiated tissues. This high rate of initiation combined with the potential of multiplying material indefinitely to regenerate thousands of plants, provides the coconut industry with a solution for their current need of quality planting material. This work provides a welcome alternative to the current coconut palm micropropagation practices that rely solely on somatic embryogenesis. Due to its high efficiency, ease of application and putative lower chance of off types this is of interest to the industry as well.

## Materials and methods

### Plant material

Zygotic embryos from six cultivars were provided by the Philippine Coconut Authority (PCA), 3 dwarf (Catigan Dwarf (CATD), Equatorial Guinea Dwarf (EGD), Malayan Yellow Dwarf (MYD)) and 3 tall (Laguna Tall (LAGT), Markham Valley Tall (MVT), West African Tall (WAT)) cultivars by accepting the terms and conditions of the Standard Material Transfer Agreement (SMTA) of the International Treaty on Plant Genetic Resources for Food and Agriculture. The use of plants in the present study complies with international, national and/or institutional guidelines. Their plant descriptors are found in the Catalogue of Conserved Coconut Germplasm^31^. The zygotic embryos were extracted at PCA from 10–11 month old coconuts after which they were locally sterilized by washing the coconut plugs (a disk, 2–3 cm diameter, of solid endosperm containing the embryo) in commercial bleach and rinsing with water^57^. After sterilization, the embryos were excised from the plugs and individually transferred to 2 mL cryotubes containing 1 ml sugarless semi-solid Y3 medium^58^, then shipped to the Laboratory of Tropical Crop Improvement Leuven, Belgium by air courier.

Previous research has shown that upon arrival an extra sterilization step needs to be included to eradicate remaining contaminations^59,60^. Therefore, the embryos were exposed to a 95% EtOH solution for 3 min, followed by 20 min washing in a 0.5% NaOCl solution containing two drops of liquid soap followed by a triple rinse with sterile demineralized water^59^.

### Medium compositions

All media are based on the Y3 medium (phytotechlab, Product nr: E2563), formulated by Eeuwens^58^ and subsequently improved by Rillo and coworkers^61^. The media, except the germination medium, all share the same basis a Y3 + vitamin mixture (exact composition available as a supplementary Table S1), 40 g/L Sucrose and 2 g/L Gelrite, but vary in AC and PGR concentrations. In our study the following culture media were used: Y40 AC (basic, 1 g/L AC); Y40 (basic); 0.1TDZ (basic, 0.1 µM TDZ); 1TDZ (basic, 1 µM TDZ); 1BA (basic, 1 µM BA); 10BA (basic, 10 µM BA); 100BA (basic, 100 µM BA); 0.1CPPU (basic, 0.1 µM CPPU); 1CPPU (basic, 1 µM CPPU); 10CPPU (basic, 10 µM CPPU). The germination medium (Y60 AC) consisted of a Y3 + vitamin mixture, 60 g/L Sucrose, 2 g/L Gelrite and 1 g/L AC. Ingredients were mixed in demineralized water, the media pH was adjusted to 6.12, with NaOH or HCl and subsequently boiled, dispensed in tubes and autoclaved.

### In vitro culture and germination of coconut embryos

Sterile embryos were transferred onto Y60 AC medium and incubated for 1 month in the dark at 24 °C. Then they were subcultured twice monthly on the same medium and incubated in the light (18 h/6 h light cycle; the light was provided by 36 W (cool white)/ 840 Lumilux fluorescent lights). After the 4^th^ month, the plantlets were transferred to Y40 AC medium and subcultured every month. At each subculture, the plants were trimmed to 3–4 cm in length and transferred to fresh medium.

### Induction of proliferation

The plantlets used for induction of proliferation were at least 4 months old. To induce meristematic proliferation, several methods and media were tested. In the ***control treatment*** plantlets were trimmed to 3–4 cm in length and transferred to the induction medium. For the “***cut protocol”*** the outer layers of leaves were cut at the base. Then, horizontal cuts were made ± 0.5 cm above and underneath the estimated location of the meristem. The remaining 1 cm long cylinder was vertically halved and both pieces were transferred upright to the induction medium, making sure that the explant is 50% submerged in the medium. For the “***meristem protocol”,*** leaf tissues were removed from the base of the coconut plantlet under a binocular microscope until its diameter was around 3 mm, leaving a cylindrical/conic shaped tissue with 3–4 leaf primordia. This cone was then transferred to the induction medium in an upright position, fully submerging the base in the medium, but leaving the meristem and leaf primordia just out of it.

Tissues were cultured at 24 °C with an 18 h/6 h light cycle provided by 36 W (cool white)/ 840 Lumilux fluorescent lights. The induction medium used was varied according the experimental set up in the following 4 paragraphs.

### The effect of cutting in the presence or absence of TDZ on induction of proliferating meristems

Plantlets of the MYD cultivar were divided over three treatments; (i) control plantlets on 1TDZ medium, (ii) plantlets treated according to the cut protocol that were transferred to Y40 medium and (iii) plantlets treated according the cut protocol and transferred to 1TDZ medium. The number of explants per treatment ranged between 141 and 144. The plants were monitored for 45 days without subculturing. Hereafter the explants showing proliferating meristems in each group were counted.

### The effect of different cytokinins or cytokinin-like PGRs on the induction of proliferating meristems

Plantlets of the MYD cultivar treated according the “cut protocol” were divided over eight different culture media: 1BA, 10BA, 100BA, 0.1TDZ, 1TDZ, 0.1CPPU, 1CPPU and 10CPPU. The number of plants per treatment ranged between 20 and 24. After 45 days the number of explants showing proliferating meristems was counted as well as the number of plantlets that had died.

### The effect of the “meristem protocol” vs “cut protocol”

Plantlets of the MYD cultivar were subjected to the **“**meristem protocol” or “cut protocol”, and transferred to 1TDZ medium. Each treatment contained 48 plantlets. After 45 days the explants showing proliferating meristems were counted.

### Proliferation of six different cultivars

The “cut protocol” in combination with 1TDZ was executed on six cultivars, 3 dwarf (CATD, EGD, MYD) and 3 tall ( LAGT, MVT, WAT) cultivars. Three repetitions of each 24 plants were used. The explants that showed proliferating clumps or died were counted after 45 days.

### Subculture of proliferating material

The proliferating tissues that were obtained in the previous experiments were isolated from their leaf base and transferred to 1TDZ medium and grown in the dark. These proliferating tissues were subcultured monthly and transferred onto fresh 1TDZ medium. For this, the clumps of meristems were divided in smaller pieces (~ 0.8 cm^2^ measured at the base) by removing non-meristematic tissues and parts that showed browning. These were then transferred one by one to new culture tubes and further maintained in the dark at 24 °C.

### Regeneration

Clumps of proliferating meristems of around 0.8 cm^2^ in size, measured at the base, were transferred to Y40 AC medium and subsequently stored in darkness at 24 °C for three months. Each month, tissues were divided in 2–5 pieces depending on the size of the regenerating meristem clump and subcultured on Y40 AC medium until only 1–6 shoots were left per tube. After three months these regenerating plantlets were transferred to light (18 h/6 h light cycle provided by 36 W (cool white)/ 840 Lumilux fluorescent lights) and further subcultured every month on Y40 AC medium until fully regenerated rooted plantlets were obtained.

During regeneration, the development of the meristems was investigated and the shoots showing browning or abnormal growth were counted.

### Scanning electron microscopy (SEM)

Some representative samples of the proliferating tissues were scanned with a 27–30 times magnification at 40 Pa (low vacuum), without any pretreatment, with SEM (JEOL JSM-6010PLUS/LV Scanning Electron Microscope with Motorized X–Y Stage).

### Statistics

The data were analysed using a contingency analysis with an alpha level of 0.05 in the statistical program JMP. If the different treatments differed significantly, subsequent analysis was done using a new contingency analysis excluding the significantly different treatments, until no significant differences were found.

The percentages were transformed using the following formula: $y = \arcsin \left( {\sqrt{\frac{x}{100}} } \right)$ to create a normal distribution. After the transformation the averages of the different cultivars were compared with each other, using an ANOVA with an alpha level of 0.05 in the statistical program JMP.

## Supplementary Information

Supplementary Information 1.

Supplementary Information 2.

## Data Availability

The datasets generated in current study, after executing the experiments described in this manuscript are available as a supplementary Data S2.
